# Pediatric Critical Care and the Climate Emergency: Our Responsibilities and a Call for Change

**DOI:** 10.3389/fped.2020.00472

**Published:** 2020-08-20

**Authors:** Gavin Wooldridge, Srinivas Murthy

**Affiliations:** ^1^St Mary's Hospital, Imperial College London, London, United Kingdom; ^2^Pediatric Critical Care, BC Children's Hospital, Vancouver, BC, Canada

**Keywords:** climate change, pediatric critical care, global health, global warming, pediatric emergencies

## Abstract

Critical care is perhaps one of the most “climate-intensive” divisions of health care. As greenhouse gas emissions continue to rise, the unprecedented threat of climate change has belatedly prompted an increased awareness of critical care's environmental impact. Within our role as pediatric critical care providers, we have a dual responsibility not only to care for children at their most vulnerable, but also to advocate on their behalf. There are clear, demonstrable effects of our worsening climate on the health of children, with the resultant increased burden of pediatric critical illness and disruption to health care systems. From increasing wildfires and their effect on lung health, to the spread of vector-borne diseases such as dengue, and the increased migration of children due to a changing climate, the effects of a changing climate are here, and we are beginning to see the changing epidemiology of pediatric critical illness. Ensuring that the effects of ongoing changes are minimized, including its future effects on child health, requires a multifaceted approach. As part of this review, we will use the Lancet Countdown on Climate Change indicators to explore the impact of pediatric critical care on climate change and the inevitable influence climate change will have on the future practice of pediatric critical care globally.

## Introduction

The world is rapidly changing. Despite the signing of the Paris Agreement in 2015 (1), greenhouse gas emissions have continued to rise (2), and if current trends continue, global warming is likely to reach 1.5°C between 2030 and 2052 (3). The Intergovernmental Panel on Climate Change Working Group 1 in 2013 stated the warming of the climate system is unequivocal and that human influence was the dominant cause (4). With limited progress toward the United Nations Sustainable Development Goal 13 (5), the current and future detrimental effect on health is felt to be irrefutable (6–9), despite the fragmented evidence base, particularly in low-income countries (10). In 2004, ~0.2% of global deaths were attributable to climate change, 85% of which were children (11). Current estimates place an additional 250,000 deaths per year between 2030 and 2050 secondary to climate change, the vast majority of which will be under the age of 5 years (12). Children are projected to bear 88% of the burden of the disease related to climate change (13, 14). With this clear direct impact on children's well-being, many consider the anthropogenic global warming a violation of the rights of future people (15).

Health professionals occupy a privileged position and are ideally placed to not only provide compelling examples of the medical consequences of climate change (16) but also inform policies that aim to mitigate its effects (17, 18). As pediatric intensive care providers, working in perhaps one of the most climate-intensive divisions of health care, we have yet to adequately prepare for the changing burden of critical illness; nor have we taken the appropriate actions to mitigate this eventuality. Our role in advocacy and as ambassadors of change, both within our institutions and across our larger communities, is essential, especially considering the substantial contribution of the health care sector to a nation's greenhouse gas emissions (19–21), the increasing awareness of the environmental impact of critical care (22–25), and the general lack of literature on climate change and health (6). We all have a duty to conserve and improve the planet for future generations. This is something the entire medical community can support, irrespective of their interpretation of the evidence related to climate change.

The direct and indirect impacts of COVID-19 on global child health are likely to be devastating for years to come. The extensive measures imposed, however, during the pandemic have led to dramatic reductions in air pollution in many major cities of the world, and the hope is that this could be a real turning point in tackling climate change. Over the coming months and years, economies will need to be reset, international travel remains restricted compared to previous, and government priorities shifted. An opportune moment therefore exists to promote, advocate, and prioritize local, national, and global efforts to tackle climate change.

**Figure 1:**
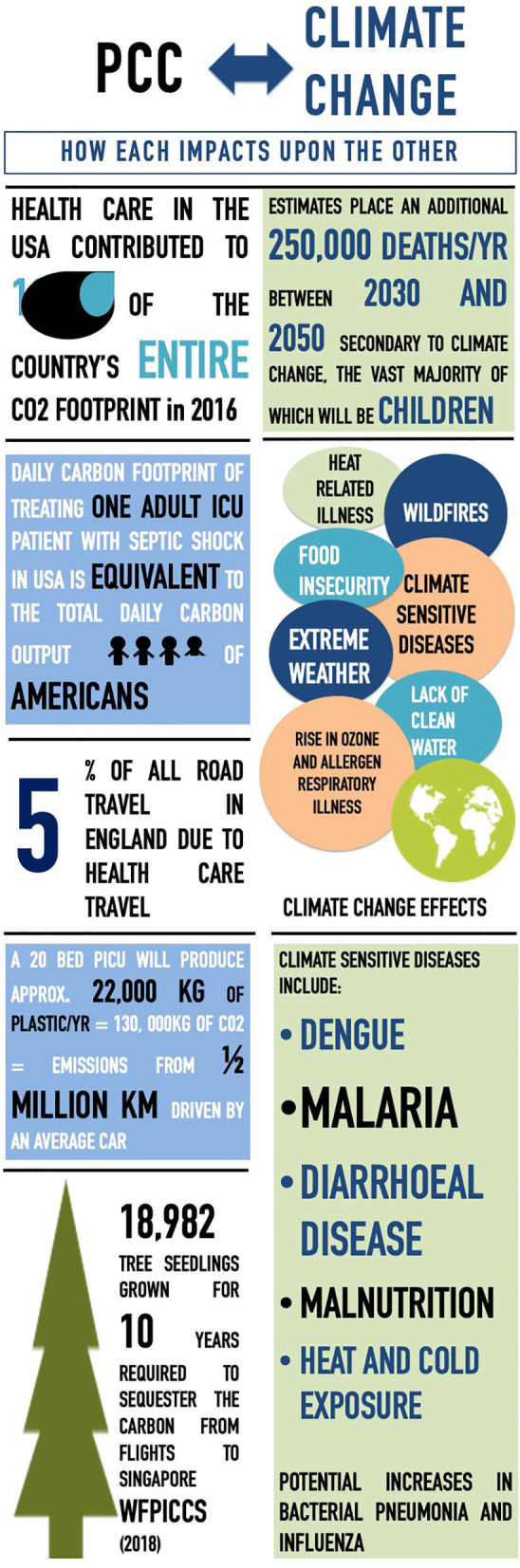
Infographic of relationship of pediatric critical care (PCC) and climate change.

The consequences of a changing climate are here, with implications for all and will only escalate in coming years. The burden, however, will fall heaviest on the vulnerable populations in low- and middle-income countries (LMICs). Compromised air quality from increasing wildfires and their effect on lung health (26), altered patterns of spread of vector-borne diseases such as dengue (27), and food insecurity and lack of clean drinking water (10), the impact of climate change upon global child health is severe. Ensuring that these effects are minimized, including its future impact on child health, requires a multifaceted approach. What can we do therefore as practitioners to not only reduce our input into the climate emergency but also respond to the already initiated changes? The “Lancet Countdown: Tracking Progress on Health and Climate Change” is a global collaboration tracking progress of a number of indicators across five domains relating to health, climate change, and their implications for national governments (6). As part of this review, we will use and highlight those Lancet indicators relevant to the care of critically ill children. The impact of pediatric critical care on climate change will be explored, and the inevitable influence climate change will have on the practice of pediatric critical care globally discussed (Figure 1).

Impact of Pediatric Critical Care on Climate Change.

- 2019 Lancet Countdown Indicator 3.9: mitigation in the health care sector, healthcare sector greenhouse gas emissions.

The health and social care sector of England represented 6.3% of the total nation's carbon footprint emissions in 2017. Almost two-thirds are accounted by supply chain emissions, one-fourth from core (including building's energy use), and one-eighth from the community including patient travel and staff commuting (21). In 2016, health care in the United States contributed to 10% of the country's entire CO_2_ footprint (19), 7% in Australia (20), and 3.5% in India (28). Globally, health care sectors were responsible for 4.4% of the global total CO_2_ emissions in 2014 (28). Significant progress can be made, however, with the implementation of legal targets and political will, as evidenced by the reduction in total health and social care emissions by 18.5% in England over the last decade (21, 29).

The total emissions per bed day in a critical care unit were calculated to be approximately equivalent to 9 kg of CO_2_ (CO_2_e) (not including heating, travel, or procurement) (30). Prior work has demonstrated the daily carbon footprint of treating one adult intensive care patient (178 kg CO_2_e) with septic shock in the United States to be equivalent to the total daily carbon output of 3.5 Americans (31). This was predominately accounted for by the heating, ventilation, and air conditioning.

### Drug Manufacturing and Its Impact

Twelve percent of health-related carbon emissions in England originate from pharmaceuticals (21), of which critical care is a significant consumer. The majority of morphine's carbon footprint, perhaps the most commonly used pediatric intensive care unit (PICU) medication, was found to derive from sterilization and packaging, with the production of every 100 mg of morphine sulfate having an emission of 204 g CO_2_, equivalent to that of the emissions of driving 1 km in an average car (32). The overall carbon footprint of pharmaceuticals is high when consideration is given to the chemicals used, the degree of refinement required in factories, transportation costs, and the fact that a large proportion go to waste (33). Potential actionable routes for individual units, hospitals, and governments to take may include an environmental rating for drugs (34) and a preference to use those medications with a lower environmental impact.

### Plastic Waste in Intensive Care Units

In the United States, health care is the second highest contributor to landfill and incinerators (24), with ~2.2 kg of waste produced per patient per day (35). A 10-bed Australian intensive care unit (ICU) at a 320-bed hospital produced 5% of total hospital waste, approximately, 44% of which was recyclable, with almost 60% of the ICU general waste suitable for recycling (36). A later audit revealed only 15% of the waste to be recyclable, with almost half (70 kg of 145 kg) actually recycled. The estimated cost was ~$13 per week (37). An average of 3.4 kg of single-use materials per patients per day (31) was used. This predominantly plastic produces, for a 20-bed PICU with 90% occupancy year-round, 22,338 kg of plastic annually, equivalent to 134,028 kg of CO_2_. For reference, this is approximately equivalent to the emissions from half a million kilometers driven by the average passenger car. Recycling itself is, however, energy-intensive, especially in a health care setting where high reprocessing standards must be met (38), and initial outlay high. Addressing this high initial cost may be difficult to justify, particularly in areas where resources are limited, and finances best diverted to actual patient care.

### Theaters

Operating theaters are an appreciable source of greenhouse gas emissions, with the use of anesthetic gases and energy consumption primarily responsible (39). Anesthetic gases represent 1.7% of the carbon footprint for all acute National Health Service organizations (21). The use of desflurane or sevoflurane from modern anesthetic machine for 1 h is the same as 230 or 30 miles traveled in a modern car, respectively (40). Progressive elimination of desflurane led to a 24% reduction in greenhouse gas emissions associated with anesthetic gas use (41).

### Travel and Transport

Telemedicine will likely play an increasing role in the care of acutely unwell children in the future (42), including promoting the virtual dissemination of knowledge, such as the well-attended World Sepsis Day virtual congress, up-skilling of local staff, and virtual meetings. It is already playing an increasing role in the fight against recent outbreaks. With health care–related travel accounting for around 5% of all road travels in England each year (21)—patient travel and staff commuting the largest contributory sources—limiting unnecessary travel and promoting work from home needs to be encouraged. There are limitations associated with the use of telemedicine in pediatric critical care, however, including the obvious inability to examine patients, privacy, and security concerns related to patient data and the initial setup cost.

An example of a direct contribution pediatric critical care has on climate change is air travel related to the World Federation of Pediatric Intensive and Critical Care Societies (WFPICCS) meetings. The carbon dioxide emissions were calculated for air travel to and from the meeting site for the nations with the 20 leading delegates. Carbon dioxide emissions were calculated according to the universally determined model, incorporating the type of airplane likely used and assuming an average occupancy of the plane. Emissions were calculated for 3,556 delegates for the three meetings (78.7% of the total 4,520 delegates). Total emissions for flying the majority of delegates to the three meetings were 760 tons for Istanbul, 856 tons for Toronto, and 1,148 tons for Singapore (Supplementary Table 1). For the purposes of large conferences, where a stated goal is to build community, virtual meetings likely do not satisfy these goals. Medical conferences will still play a role for advancing our field—research presented at meetings in face-to-face conversations is cited more highly than that which has not (43), and scientific progress in our field has direct impact on the lives of sick children, including those affected by a changing climate. The interpersonal relationships built through face-to-face contact are likely irreplaceable and not quantifiable (44).

## Limiting our Climate Change Contribution

The pediatric critical care community needs to decide how best to lead the health care sector in advocacy and change (Table 1). As stewards of the health of children, our professional careers are directly linked to the future health of the planet, and we have an obligation to serve as role models for other fields. There is a compelling argument for the health sector to lead by example in implementing mitigation measures (45). A number of medical organizations have either passed resolutions calling for financial divestment from fossil fuel companies (46), produced statements on environmental sustainability (47), or written open letters to governments (48). Numerous staff surveys have revealed a willingness to engage in measures to reduce their environmental impact (21, 49, 50). The ability to reduce our dependence on plastic within in critical care is crucial, and we should all encourage recycling and safe reuse of equipment where practical. Local council members or hospital board members can be petitioned to ensure food and other purchases are sustainably sourced (24, 51), and that new building specifications abide by the World Health Organization (WHO) framework for climate-resilient health systems (52) and are climate-friendly (51). Promoting climate change as a health issue may have greater political and public resonance (53) and lead to better staff engagement.

**Table 1:** Opportunities to reduce emissions in pediatric critical care.

**Areas of improvement in PICU**	**Opportunities for change**
**CLINICAL CARE**	
• Medications	• Reuse, limit amount discarded. • Preference for those less harmful to environment • Environmental rating for drugs [34] • Become “critical consumers” and purchasers ∘ Make inquiries over environmental costs, possible harmful effects, sustainability, and the existence of alternatives
• Consumables, including single use plastic	• Reduce, reuse, and recycle [24] ∘ Requires separating waste into infectious and non-infectious
• Telemedicine	• Promote increased use • Limit unnecessary travel and transfers
• Waste and linen	• Promote recycling ∘ Increase availability of recycling bins • Use minimum temperature “allowable” to clean linen
• Equipment	• Advocate for reprocessing of old equipment • Ensure energy efficient • Ensure off when not in use
• Organizations	• Petition for financial divestment from fossil fuels • Encourage further collaboration between units • Research into future effects of climate change
**ADMINISTRATIVE**	
• Paper	• Limit printing, encourage electronic storage and data transfer
• Procurement and purchasing	• Advocate for sustainably sourced, explore environmental costs, • Become “critical consumers”
• Staff	• Facilitate working from home for administrative staff
**BUILDING**	
• Energy consumption (lighting, heating, electrical appliances)	• Advocate for ∘ Energy efficient lightning ∘ Sensors to control lighting ∘ Use of energy meters ∘ Temperature controls • Ensure equipment off and not on standby
• Water	• Advocate for timers on water taps
• Food	• Advocate for sustainably sourced food • Promote limitation of food waste
• Specifications	• Ensure meet the necessary environmental regulations • Ensure climate friendly
**PERSONAL**	
• Commute to work	• Promote emission free transport • Promote health benefits of walking or cycling to work • Online carbon footprint calculators can highlight areas for individual improvement
• Conferences/meetings	• Encourage use of videoconferencing
• Lifts	• Promote use of stairs
• Drink and food containers	• Promote use of reusable products
**ORGANIZATION**	
• Pediatric critical care societies	• Petition for financial divestment from fossil fuels • Encourage further collaboration between units • Research into future effects of climate change: ∘ on provision of critical care ∘ future epidemiology of pediatric critical illness ∘ methods of reducing carbon footprint and environmental impact

As the costs, both monetary and environmental, of fossils fuels are likely to increase in the coming decades, as too will the costs to the health care sector (38), a significant consumer. The financial impact of climate-sensitive events is also substantial (54). Discussions at a local level will have to evaluate a multitude of factors including a cost-benefit analysis of the initial high startup expense and the long-term return on investment. When compared to the cost savings from the health and social benefits of these policies, however, including combatting health inequalities that will be exacerbated by climate change, the long-term cost of mitigation efforts required to stabilize climate change is thought to be relatively small over the long term (38, 45).

### Impact of Climate Change on Pediatric Critical Care

At least seven of the Lancet Countdown indicators are implicated in the care of the critically ill child.

- 2019 Lancet Countdown Indicator 1.1: health and heat
- 2019 Lancet Countdown Indicator 1.2: health and extreme weather events
- 2019 Lancet Countdown Indicator 1.3: global health trends in climate-sensitive diseases
- 2019 Lancet Countdown Indicator 1.4: climate-sensitive infectious diseases
- 2019 Lancet Countdown Indicator 1.5: food security and under nutrition
- 2019 Lancet Countdown Indicator 3.3.1: exposure to air pollution in cities
- 2019 Lancet Countdown Indicator 3.3.2: premature mortality from ambient air pollution by sector

**Figure 2:**
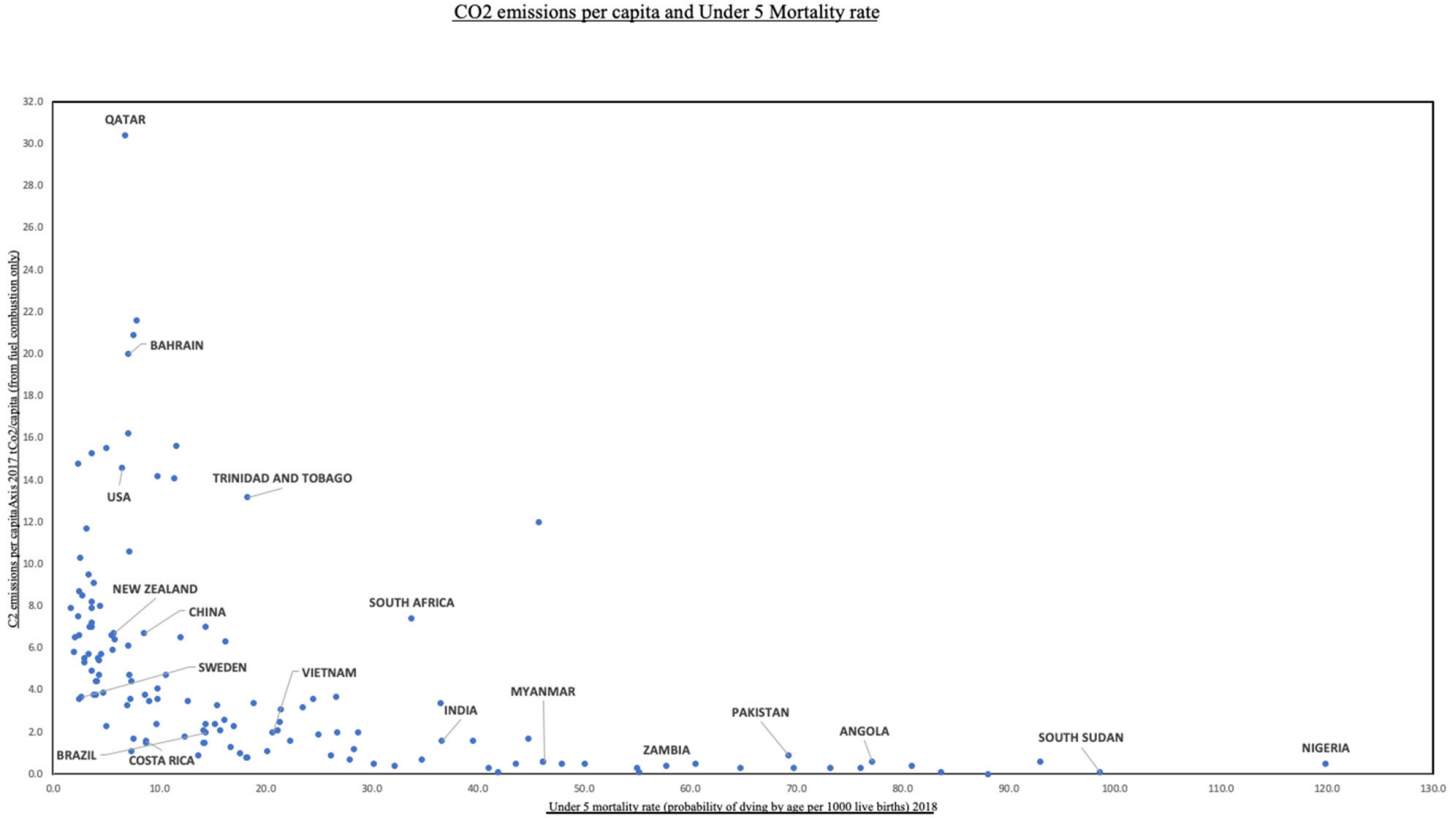
CO_2_ emissions per capita and under-5 mortality rate. Data from World Bank (https://data.worldbank.org/).

The dire effects of the climate emergency on global child health will impact pediatric critical care units worldwide. The degree to which climate change will alter the current daily running of an ICU is, however, unclear. With limited ability and infrastructure to mitigate the impact of climate change, those working in an LMIC setting will undoubtedly face the greatest challenges, while being the ones least responsible for its causes (Figure 2). The United Nations Environment found that nearly a quarter of all deaths globally in 2012 could be attributed to modifiable environmental risks, with a greater portion occurring in populations in a vulnerable situation and in developing countries (55).

In Sub-Sahara Africa, total annual expenditure on health is commonly under US $25 per capita and often <3% of the gross domestic product, compared to 8.5% in the United States (56). With limited budgets and challenges accessing international climate finance for health, many nations do not have a national health and climate change strategy, let alone the means to implement it (57). Furthermore, the number of intensive care beds as a percentage of hospital beds is ~1.5% in LMICs compared to 2.5 to 9% in high-income countries (58). Concerns extend to dealing with increased and unexpected acute demand, blackouts caused by disruption to the power supply, increased air conditioning use if present, and coping with extreme heat (59). As the link between climate change and the increasing frequency of natural disasters and variable rainfall continues to be explored, the impact of both acute and longer-term weather events on medical care and societies in LMICs will be devastating. Loss of medical records, damage to water supplies and sanitation, and interruption to medical supply chains from extreme weather events are real and pressing fears (60, 61). In addition, health access for climate refugees may be restricted, and staff and patients experience great difficulties in physically accessing the hospital (62) secondary to natural disasters. At present, 1.6 billion people live in places where protracted crises (including famine, conflict, drought, and population displacement), and weak health care services leave them without access to basic care (63).

#### Heat and Air Pollution

An inability to care for oneself, the presence of a chronic illness, and certain medications (including diuretics) are all risk factors for heat illness (64, 65). These are clearly relevant to a high proportion of children who are unable to modify their exposure risk and are at a greater risk of dehydration (66). Those with cardiac, respiratory, or renal disorders are at increased risk and will form an increasing proportion of the pediatric critical care population as non-communicable disease rates rise, particularly with a lack of air conditioning for many children in LMICs (65).

With a rise in ambient temperatures and wildfires, levels of ozone, allergens, and other pollutants will increase, potentially triggering acute asthma exacerbations (66–68). Nine out of 10 people already breathe polluted air every day, and in 2019, the WHO deemed air pollution as the greatest environmental risk to health (63). Working on action plans prior to discharge is therefore paramount and may include staying indoors and avoiding unnecessary outdoor activity on poor air quality days (67). Advocating for widespread dissemination of information on air quality, exploring non-polluting methods of inhaler delivery and advance planning for extreme weather events are essential.

#### Changing Weather and Infectious Diseases

A number of diseases have been deemed climate-sensitive including dengue, malaria, diarrhea, heat and cold exposure, and protein-energy malnutrition. Deaths from malaria, diarrheal disease, and malnutrition are all currently decreasing (6). However, because of changes in vector distribution and environmental suitability (6, 69), vector-borne diseases are emerging in previously unaffected regions. Of particular relevance to pediatric critical care is the rapid increase in dengue fever (6), especially in South East Asia. The incidence and severity of respiratory infections may alter over the coming years, with potential increases in bacterial pneumonia and influenza and a reduction in respiratory syncytial virus during the milder winters (70). Managing this population of high-risk, critically unwell individuals with little physiological reserve in settings with strained health systems due to acute weather events, migration, or conflict will be challenging and require a global coordinated response. In addition to the potentially disrupted medical and utilities infrastructures, a compromised vaccination program and restricted medication availability are all prime breeding grounds for epidemics (71). Higher temperatures and humidity will lead to an increased risk of food and water borne diseases (17).

## Responding to Health Impacts of Climate Change

Pediatric critical care practitioners should anticipate a number of variations in their workload as climate change takes a progressive hold. Indeed, a large survey of American Thoracic Society members indicated many had already observed health impacts of climate change among their patients (50), and other reports reveal its current adverse effect on health care and health care systems (8, 9, 54, 72). A rise in demand, with a predominance of heat-related disorders, respiratory illness, and climate-sensitive diseases, interspersed with the direct and indirect effects of extreme weather events on health and infrastructure, will challenge us all (Table 2).

**Table 2:** Anticipated changes to pediatric critical care.

**Climate change effect**	**Potential impact on pediatric critical care**
Heat	• Increase in heat-related illness ∘ Increased admissions, potential increased renal replacement therapy secondary to rhabdomyolysis • Increase in air conditioning use ∘ Increase greenhouse gas emission, higher energy bills • Impact on staff welfare ∘ Increased sick leave • Increase in pneumonia incidence ∘ Increased admissions
Extreme weather events	• Increase in drowning and trauma • Rapid surges in demand and need to scale up capacity • Difficulty in both staff and patients accessing health facility • Disruption to supply chains, infrastructure, and buildings • Disruption to electrical and water supplies • Need for coordinated triage systems
Food insecurity and limited clean drinking water and sanitation	• Higher proportion with under nutrition leading to lifelong health effects • More research required on the care of the critically ill malnourished child in PICU
Climate-sensitive diseases	• Increase in severe malaria, dengue fever, and diarrheal disease • Increasing incidence in previously unaffected areas—training required for those unfamiliar
Staff physical and mental health	• Coping with acute disasters, high demand, potential rationing and limited resources • Recruitment and retaining may prove challenging in climate affected areas
Climate refugees	• Historically have reduced access to health services • May have limited local language skills and access to their health documents • Limited funds to pay for health care
Conflict	• Increased migration • Increased risk of epidemics • Increased trauma • Impact on staff welfare • Distribution to infrastructure and supply chains
Air pollution, including wildfires	• Increase in acute respiratory illness, asthma and severe burns • Need for local authorities to publish frequent air quality data, in order to anticipate PICU demand

Critical care skills and experience are relevant to each of the WHO's six common “building blocks” for health systems, although importantly critical care is not limited to the ICU. The timely provision of antibiotics to a child with pneumonia is every bit as critical as establishing a periarrest patient on extracorporeal life support (73), and the occurrence of the former will be of vital importance to the vast majority affected by climate change.

Our role in the anticipation and response to current and future health impacts of climate change is essential, as is our involvement in the development of climate-resilient health systems. We have a powerful voice to advocate locally and nationally for change, not only to raise awareness but also to promote partnerships, encourage scientific research, and aid the strengthening of health systems (57). As a field, we need to adapt to this emerging threat and work cohesively with other organizations to first formally recognize the urgent need for action and, second, instigate partnerships and plans to mitigate its effect. We should ensure that staff, our units, hospitals, and local authorities are all aware of the likely impact climate change will have on us all over the coming years and the increased demand on critical care resources and services that will ensue. As a collaboration, tracking PICU-specific Lancet Countdown metrics, both at a local and national level, would inform health care providers and policy holders of progress made toward a sustainable and carbon neutral future, ultimately keeping us accountable to the critically ill children worldwide we strive to care for.

## Conclusion

Climate change will have profound, acute, and long-term detrimental effects on the children of today and in the future. As members of the pediatric critical care community, we have a duty to defend and improve upon global child health. Our likely presence at the climate frontier, especially during acute events, demands that we play a pivotal role in mitigating and adapting to the effects of climate change on the critically ill child. Promoting research into climate change and its complex interaction with health is vital, expanding the evidence base and allowing governments and organizations to make informed choices regarding mitigation strategies. Critical care–specific climate studies would allow us as a profession to mitigate, prepare, advocate, and inform future policies. High initial short-term mitigation costs will likely be outweighed by future health benefits. How the current COVID-19 pandemic affects the progression of climate change is as yet unknown, but the reset to society provides an opportune moment to reinvigorate efforts and focus minds once more.

## Data Availability Statement

The original contributions presented in the study are included in the article/Supplementary Material, further inquiries can be directed to the corresponding author/s.

## Author Contributions

GW and SM were involved in article conception. GW was responsible for the first draft of the manuscript. Both were involved in drafting and editing the final manuscript.

## Conflict of Interest

The authors declare that the research was conducted in the absence of any commercial or financial relationships that could be construed as a potential conflict of interest.
