# Supplementary material for: Pediatric Critical Care and the Climate Emergency: Our Responsibilities and a Call for Change
Source: Front Pediatr. 2020 Aug 20;8:472. doi: 10.3389/fped.2020.00472 (PMC7468581; doi:10.3389/fped.2020.00472)
Supplement: Supplementary file 1 [file Table_1.docx]

**Supplementary Table 1**: Total emissions for travel to recent WFPICCS meetings for delegates from top 20 countries by number of attendees

| Singapore 2018 (n=1440) | | | Toronto 2016 (n=1536) | | | Istanbul 2014 (n=1544) | | |
| --- | --- | --- | --- | --- | --- | --- | --- | --- |
| Country | **Percent of total delegates (%)** | **Tonnes of CO_2_** | **Country** | **Percent of total delegates (%)** | **Tonnes of CO_2_** | **Country** | **Percent of total delegates (%)** | **Tonnes of CO_2_** |
| UK | 11.0 | 276.6 | USA | 22.4 | 31 | Turkey | 12.5 | 0 |
| Australia | 9.2 | 122.4 | Canada | 20.8 | 0 | UK | 10.9 | 71 |
| USA | 8.9 | 286.7 | UK | 12.1 | 169.3 | USA | 10.8 | 195.9 |
| Singapore | 7.7 | 0 | Brazil | 5.3 | 75.3 | Australia | 5.2 | 176.6 |
| India | 6.0 | 53.1 | Australia | 5.0 | 174.8 | Canada | 5.1 | 93.6 |
| China | 5.0 | 46.8 | India | 2.9 | 74.8 | Brazil | 3.3 | 47.4 |
| Philippines | 4.1 | 20.7 | Japan | 2.6 | 60.4 | China | 2.9 | 46.3 |
| Indonesia | 4.0 | 20.3 | Netherlands | 2.1 | 29.0 | Saudi Arabia | 2.3 | 13 |
| Canada | 3.0 | 93.3 | Switzerland | 1.6 | 23.7 | India | 2.3 | 24.1 |
| Thailand | 2.6 | 8 | Mexico | 1.5 | 11.0 | Germany | 1.9 | 8.1 |
| Japan | 2.2 | 25 | China | 1.2 | 29.4 | Netherlands | 1.9 | 9.6 |
| Malaysia | 2.2 | 1.2 | Turkey | 1.2 | 21.6 | Sweden | 1.9 | 9.6 |
| Netherlands | 2.1 | 46.2 | France | 1.1 | 15 | Switzerland | 1.7 | 6.8 |
| Hong Kong | 2.0 | 11.0 | Israel | 1.1 | 23.1 | Indonesia | 1.6 | 34.5 |
| Brazil | 1.6 | 56.8 | Singapore | 1.0 | 32.56 | Italy | 1.5 | 4.6 |
| New Zealand | 1.6 | 28.3 | Italy | 0.9 | 14.6 | Israel | 1.4 | 3.6 |
| Turkey | 1.2 | 21.6 | South Africa | 0.8 | 25.3 | Lebanon | 1.3 | 2.8 |
| Cambodia | 1.2 | 2.9 | Germany | 0.8 | 12.1 | Romania | 1.2 | 1.3 |
| South Korea | 1.0 | 9.5 | South Korea | 0.8 | 18.6 | Norway | 1.2 | 6.8 |
| South Africa | 1.0 | 17.6 | Chile | 0.8 | 15.1 | Libya | 1.2 | 4.3 |
|  | **77.6** |  |  | **86.1** |  |  | **72.3** |  |
| Total calculated emissions (tonnes) |  | **1148** |  |  | **856** |  |  | **760** |
